# Supplementary material for: The lateral stress profile of fluid lipid membranes as revealed by the diffuse interface approach
Source: Biophys J. 2025 Aug 5;124(18):2984–94. doi: 10.1016/j.bpj.2025.07.041 (PMC12709251; doi:10.1016/j.bpj.2025.07.041)
Supplement: Document S1. Supporting material [file mmc1.pdf]

Biophysical Journal, Volume *124*

Supplemental information

The lateral stress profile of fluid lipid membranes as revealed by the diffuse interface approach

Matteo Bottacchiari, Mirko Gallo, Marco Bussoletti, and Carlo Massimo Casciola

SUPPLEMENTARY MATERIAL
The lateral stress profile of fluid lipid membranes as revealed by the diffuse interface approach

Matteo Bottacchiari,[1,2] Mirko Gallo,[2] Marco Bussoletti,[2] and Carlo Massimo Casciola[2,*]

[1]*Department of Basic and Applied Sciences for Engineering, Sapienza University of Rome*
[2]*Department of Mechanical and Aerospace Engineering, Sapienza University of Rome*

(Dated: December 4, 2024)

INTERMEDIATE STEPS FOR EQ. (21)

$$
\begin{aligned}
E_{\mathrm{B}}[\phi] = {} & 4\pi k \frac{3}{\sqrt{2}} \int_0^{+\infty} \frac{f_0'^2\left(\frac{1/2-\bar{r}}{\lambda}\right)}{\lambda} \left(\frac{1}{\bar{r}} - \bar{m}\right)^2 \bar{r}^2 d\bar{r} + O(\lambda) \approx \\
& 4\pi k \frac{3}{\sqrt{2}} \int_0^{+\infty} \frac{f_0'^2\left(\frac{1/2-\bar{r}}{\lambda}\right)}{\lambda} \left(1 - 2\bar{m}\bar{r} + \bar{m}^2\bar{r}^2\right) d\bar{r} + O(\lambda) = \\
& 4\pi k \frac{3}{\sqrt{2}} \int_{-\infty}^{1/(2\lambda)} f_0'^2\left(z^*\right) dz^* \\
& - 8\pi k \bar{m} \frac{3}{\sqrt{2}} \int_{-\infty}^{1/(2\lambda)} f_0'^2\left(z^*\right) \left(\frac{1}{2} - \lambda z^*\right) dz^* \\
& + 4\pi k \bar{m}^2 \frac{3}{\sqrt{2}} \int_{-\infty}^{1/(2\lambda)} f_0'^2\left(z^*\right) \left(\frac{1}{2} - \lambda z^*\right)^2 dz^* + O(\lambda) \approx \\
& 4\pi k \frac{3}{\sqrt{2}} \int_{-\infty}^{+\infty} f_0'^2\left(z^*\right) dz^* - 4\pi k \bar{m} \frac{3}{\sqrt{2}} \int_{-\infty}^{+\infty} f_0'^2\left(z^*\right) dz^* \\
& + 4\pi k \bar{m}^2 \frac{3}{4\sqrt{2}} \int_{-\infty}^{+\infty} f_0'^2\left(z^*\right) dz^* = \\
& 4\pi \int_{-\infty}^{+\infty} z^2 \frac{3k}{\sqrt{2}\epsilon^3} \left[f_0''^2\left(-\frac{z}{\epsilon}\right) + f_0'\left(-\frac{z}{\epsilon}\right) f_0'''\left(-\frac{z}{\epsilon}\right)\right] dz \\
& + 4\pi D_{ve} \int_{-\infty}^{+\infty} -z \frac{3}{\sqrt{2}} \frac{2mk}{\epsilon^2} f_0'\left(-\frac{z}{\epsilon}\right) f_0''\left(-\frac{z}{\epsilon}\right) dz \\
& + \pi D_{ve}^2 \int_{-\infty}^{+\infty} -m^2 \frac{3}{\sqrt{2}} \frac{k}{\epsilon} f_0\left(-\frac{z}{\epsilon}\right) f_0''\left(-\frac{z}{\epsilon}\right) dz = \\
& 4\pi \int_{-\infty}^{+\infty} z^2 \, s_{\mathrm{B2}}(z) \, dz + 4\pi D_{ve} \int_{-\infty}^{+\infty} z \, s_{\mathrm{B1}}(z) \, dz \\
& + \pi D_{ve}^2 \int_{-\infty}^{+\infty} s_{\mathrm{B0}}(z) \, dz \, ,
\end{aligned}
$$

* carlomassimo.casciola@uniroma1.it

INTERMEDIATE STEPS FOR EQ. (23)

$$
\begin{aligned}
E_{\mathrm{G}}[\phi] = & k_{\mathrm{G}}\frac{35}{8\sqrt{2}}4\pi\int_{0}^{+\infty}\frac{f_0'^4\left(\frac{1/2-\bar{r}}{\lambda}\right)}{\lambda}\,d\bar{r}+O(\lambda^2)= \\
& k_{\mathrm{G}}\frac{35}{8\sqrt{2}}4\pi\int_{-\infty}^{1/(2\lambda)} f_0'^4(z^*)\,dz^* \;+\; O(\lambda^2)\;\approx \\
& k_{\mathrm{G}}\frac{35}{8\sqrt{2}}4\pi\int_{-\infty}^{+\infty} f_0'^4(z^*)\,dz^* \;= \\
& k_{\mathrm{G}}\frac{35}{8\sqrt{2}}4\pi\int_{-\infty}^{+\infty}\frac{z^{*2}}{2}\left[12f_0'^2(z)f_0''^2(z)+4f_0'^3(z)f_0'''(z)\right]dz^*= \\
& 4\pi\int_{-\infty}^{+\infty} z^2\,s_{\mathrm{G}}(z)\,dz\,,
\end{aligned}
$$

INTERMEDIATE STEPS FOR EQ. (26)

$$
\begin{aligned}
E_{\mathrm{T}}[\phi] = & \gamma\,D_{ve}^2\,\frac{3}{2\sqrt{2}}\,4\pi\int_{0}^{+\infty}\frac{f_0'^2\left(\frac{1/2-\bar{r}}{\lambda}\right)}{\lambda}\bar{r}^2\,d\bar{r}+O(\lambda^2)= \\
& \gamma D_{ve}^2\frac{3}{2\sqrt{2}}4\pi\int_{-\infty}^{1/(2\lambda)} f_0'^2\,(z^*)\left(\frac{1}{2}-\lambda z^*\right)^2 dz^*+O(\lambda^2)\approx \\
& \gamma\,\pi D_{ve}^2\,\frac{3}{2\sqrt{2}}\int_{-\infty}^{+\infty} f_0'^2\,(z^*)dz^* = \\
& \pi D_{ve}^2\int_{-\infty}^{+\infty} -\gamma\frac{3}{2\sqrt{2}}\frac{1}{\epsilon}f_0\left(-\frac{z}{\epsilon}\right)f_0''\left(-\frac{z}{\epsilon}\right)dz= \\
& \pi D_{ve}^2\int_{-\infty}^{+\infty} s_{\mathrm{T}}(z)\,dz\,.
\end{aligned}
$$
