# Supplementary material for: The lateral stress profile of fluid lipid membranes as revealed by the diffuse interface approach
Source: Biophys J. 2025 Aug 5;124(18):2984–94. doi: 10.1016/j.bpj.2025.07.041 (PMC12709251; doi:10.1016/j.bpj.2025.07.041)
Supplement: Document S2. Article plus supporting material [file mmc2.pdf]

Biophysical Journal
Article

Biophysical Society

The lateral stress profile of fluid lipid membranes as revealed by the diffuse interface approach

Matteo Bottacchiari,[1,2] Mirko Gallo,[2] Marco Bussoletti,[2] and Carlo Massimo Casciola[2,*]
[1]Department of Basic and Applied Sciences for Engineering, Sapienza University of Rome, Rome, Italy and [2]Department of Mechanical and Aerospace Engineering, Sapienza University of Rome, Rome, Italy

ABSTRACT The distribution of lateral stress within a lipid bilayer is of fundamental biological interest as it regulates membrane-protein interactions, with a particular impact on mechanosensitive channels. At the same time, the lateral stress profile is thermodynamically related to the elastic constants governing the macroscopic behavior of membrane vesicles. Therefore, it is tempting to try to understand how macroscopic elastic constants affect stresses within the membrane, and vice versa. It has recently been shown that a diffuse interface description of the membrane captures key features of both the macroscopic scale and the few-nanometer scale of the bilayer thickness. The approach provides the lateral stress profile as a function of the macroscopic elastic constants, whereas, usually, the reverse procedure is used in molecular dynamics simulations to extract the constants from the measured profile. Here, the complete expression of the lateral stress profile of the diffuse interface is derived, also taking into account the case of membranes under tension. The profile turns out to be the superposition of several contributions that depend on the various elastic constants. We show that tension affects the Gaussian modulus of the bilayer, in agreement with independent arguments. The approach provides a correction to the second moment of the lateral stress comparable to that based on the monolayer-bilayer consistency relation. Finally, we discuss changes in the lateral stress profile due to the insertion of molecules within the membrane. Such changes play a crucial role in membrane-protein interactions, as they influence part of the work required to gate channels.

SIGNIFICANCE Connecting the distribution of stresses within the membrane with the elastic constants governing the large-scale behavior of vesicles is of fundamental importance for understanding membrane-protein interactions through a few experimentally accessible parameters. The diffuse interface approach provides an analytical expression for the lateral stress profile as a function of the elastic constants, permitting investigation of how their variation affects the stress distribution, which provides part of the work that must be done to gate channels. Importantly, insertion of molecules within the bilayer modifies the elastic constants, and it has been proposed that amphipathic molecules, such as antipsychotics or local anesthetics, may affect protein function mainly through membrane-mediated activity.

INTRODUCTION

A fluid lipid bilayer membrane is an essential component of viruses, subcellular organelles, cell nucleus, and the cell itself. Among the many functions performed by the membrane in various biological processes, there is the direct interaction with membrane proteins through mechanical stresses, which play a role that is not yet fully understood.

The macroscopic behavior of fluid lipid membranes is successfully described by the elastic Canham-Helfrich model, which assigns a curvature-dependent energy density to the bilayer mid-surface Γ, thus neglecting the 5-nm thickness of the membrane. Denoted by M and G, the local mean and Gaussian curvature of the bilayer mid-surface, respectively, the Canham-Helfrich energy reads

$$E_{CH}[\Gamma] = \gamma \int_\Gamma dS + 2k \int_\Gamma (M - m)^2 dS + k_G \int_\Gamma G\, dS, \quad (1)$$

where γ is the tension to which the bilayer is subjected and k and k_G are the elastic constants that characterize the membrane. Typically, the bending rigidity k is roughly 20 k_BT (1,2), a value that can easily be doubled in the presence of cholesterol (2,3). The bilayer spontaneous curvature m sets the preferred curvature of the membrane, modeling the asymmetry, e.g., a compositional difference between the two leaflets, which may be due to the presence of

Submitted December 4, 2024, and accepted for publication July 31, 2025.
*Correspondence: carlomassimo.casciola@uniroma1.it
Editor: Michael Kozlov.
https://doi.org/10.1016/j.bpj.2025.07.041
© 2025 The Author(s). Published by Elsevier Inc. on behalf of Biophysical Society.
This is an open access article under the CC BY license (http://creativecommons.org/licenses/by/4.0/).

different lipids and other factors, like the unilateral adsorption of proteins (3). Here, the mean curvature is assumed to be positive, as the membrane bulges toward the exterior of the vesicle. As regards the Gaussian modulus k_G, it is expected to be in the range $-k$ to $-0.7k$ (4,5). Thermodynamics provides the connection between the elastic constants of the two-dimensional surface Γ and the lateral stress distribution across the membrane thickness (6), induced by the lipids' amphiphilic structure. According to its definition, the lateral stress profile, $s(z)$, is the difference between the tangential and normal stress, where z is the coordinate normal to the membrane and $z = 0$ the bilayer mid-surface Γ. The connection between the Canham-Helfrich elastic constants and the lateral stress is obtained through the moments of $s(z)$,

$$\mathbb{P}_i = \int_{-\infty}^{+\infty} z^i s(z) dz, i = 0, 1, 2. \quad (2)$$

In fact, it can be shown that (6)

$$\Sigma = \mathbb{P}_0^0, \quad (3a)$$

$$-2km = \mathbb{P}_1^0 + \frac{1}{2}\left(\frac{\partial \mathbb{P}_0^s}{\partial M}\right)_{T,\mu}^0, \quad (3b)$$

$$k_G + 2k = \mathbb{P}_2^0 + 2\left(\frac{\partial \mathbb{P}_1^s}{\partial M}\right)_{T,\mu}^0 + \frac{1}{2}\left(\frac{\partial^2 \mathbb{P}_0^s}{\partial M^2}\right)_{T,\mu}^0, \quad (3c)$$

where T and μ denote constant temperature and chemical potential, s refers to a spherical vesicle, and the superscript 0 is an evaluation at the planar interface limit. The derivatives are often overlooked in the literature but should solve (6,7) the ambiguity in determining the elastic constants due to the nonuniqueness of the local stress tensor (8). These relations hold for a spherical vesicle, which is the case we consider in this work. The zeroth moment provides the total tension $\Sigma = \gamma + \widehat{\Sigma}$, where $\widehat{\Sigma} = 2km^2$ is the bilayer spontaneous tension (9). Often, in molecular dynamics simulations (5), $s(z)$ is evaluated to extract the elastic constants through Eqs. 3a, 3b, and 3c. It is worth noticing that in the above relations (Eqs. 3a, 3b, and 3c), derivatives are taken at constant chemical potential, which, e.g., assumes the definition of k as a second derivative of the grand potential at constant μ, which is different from the standard definition that assumes constant number of lipids (10). Hu et al. (5) mentioned the absence of derivatives in their discussion on the implausible values of the Gaussian modulus obtained through the evaluation of $\mathbb{P}_2^0$. To the best of our knowledge, derivatives analogous to those of Eqs. 3a, 3b, and 3c but taken at constant number of lipids have never been investigated numerically. At a constant number of lipids, the derivatives of the chemical potential should also enter the equations. Based on standard continuum approaches (11), the derivatives at a constant number of lipids entering the equation for the first moment may be expected to cancel out, whereas those associated with the second moment are expected to cancel out the term $2k$. It should be stressed, however, that the results to be discussed in this work are obtained completely independently of Eqs. 3a, 3b, and 3c or analogs.

The Canham-Helfrich model treats the bilayer as an infinitely thin (sharp) surface due to the scale separation between the membrane thickness and the characteristic size of membrane vesicles, $D_{ve} = \sqrt{A/\pi}$, where A is the surface area of the vesicle. However, there are cases—such as that of topological transitions—in which both the large scale of the vesicle and the small scale of the membrane thickness are simultaneously important. For this reason, we introduced a diffuse interface description of lipid bilayer vesicles (12) that recovers the Canham-Helfrich model in the limit of small thickness/vesicle size ratios. Recently, it has been shown that the approach captures key features of the membrane interface behavior while maintaining the macroscopic description of the Canham-Helfrich model, allowing simulations that contain both the vesicle and membrane scales (13). In particular, the diffuse interface approach provides a coarse-grained version of the lateral stress profile. We stress that a closed-form expression of the (coarse-grained) lateral stress follows as a function of the elastic constants, yielding a new top-down point of view.

In this work, we calculate the complete expression of the diffuse interface lateral stress profile, also considering the case of membranes with nonzero γ. We show that the overall lateral stress is the superposition of different contributions depending on the various elastic constants. Moreover, we find that tension affects the Gaussian modulus, in accordance with independent arguments (5,14). We will show that the diffuse interface provides a correction to $\mathbb{P}_2^0$ equal to $z_D^2\Sigma$, with z_D that is approximately $2/3$ of half of the diffuse interface width (membrane thickness). In the literature, the monolayers' neutral plane is estimated to be near the hydrophilic/hydrophobic interface, namely at about $2/3$ of the thickness of the monolayer (10,15). Eventually, we will discuss the results in the context of membrane-protein interaction.

MATERIALS AND METHODS

The diffuse interface approach

In this section, we briefly recall the mathematical aspects of the diffuse interface description, which is based on a Ginzburg-Landau type of free energy $E[\phi]$ (12). The phase-field $\phi(\mathrm{x})$ is a smooth function defined everywhere in a domain $\Omega \subseteq \mathbb{R}^3$, which takes values in the range $[-1, 1]$. In particular, $\phi = -1$ in the outer environment of the vesicle, whereas $\phi = +1$ in the inner environment. The small transition layer between these two values represents the membrane interface, whose width is determined by a small parameter ϵ. Thus, the sharp interface of the Canham-Helfrich

model is here replaced by a diffuse layer, introducing an additional length scale. The isosurface $\phi = 0$ will be identified with the bilayer mid-surface Γ of the Canham-Helfrich model. The integral-type functional $E[\phi]$ reads

$$E[\phi] = E_{\mathrm{B}}[\phi] + E_{\mathrm{G}}[\phi], \tag{4}$$

where

$$E_{\mathrm{B}}[\phi] = k\,\frac{3}{4\sqrt{2}}\,\epsilon \int_{\Omega} \Psi_{\mathrm{B}}^2\, dV, \tag{5}$$

$$\Psi_{\mathrm{B}} = \nabla^2\phi - \frac{1}{\epsilon^2}\left(\phi^2 - 1\right)\left(\phi + \sqrt{2}\,\epsilon m\right) \tag{6}$$

represents the bending term of the Canham-Helfrich energy (second term of Eq. 1) and

$$E_{\mathrm{G}}[\phi] = k_{\mathrm{G}}\,\frac{35}{16\sqrt{2}}\,\epsilon^3 \int_{\Omega} \Psi_{\mathrm{G}}\, dV, \tag{7}$$

$$\begin{aligned}\Psi_{\mathrm{G}} = &\frac{\nabla|\nabla\phi|^2\cdot\nabla|\nabla\phi|^2}{2} - \left(\nabla|\nabla\phi|^2\cdot\nabla\phi\right)\nabla^2\phi \\ &+ |\nabla\phi|^2\left[\left(\nabla^2\phi\right)^2 + \nabla\phi\cdot\nabla\nabla^2\phi - \frac{\nabla^2|\nabla\phi|^2}{2}\right]\end{aligned} \tag{8}$$

represents the Gaussian term (third term of Eq. 1). The bending term $E_{\mathrm{B}}[\phi]$ was initially introduced by Du et al. (16–18) and subsequently used by several authors (19–23), whereas the Gaussian contribution $E_{\mathrm{G}}[\phi]$ was recently introduced in (12). Following the calculations reported in such work (12), the phase field is assumed to satisfy the ansatz

$$\phi(\mathbf{x}) = f\left(\frac{d(\mathbf{x})}{\epsilon}\right), \tag{9}$$

where $d(\cdot)$ is the signed distance function from the bilayer mid-surface Γ of the Canham-Helfrich model. The signed distance is such that the inward-pointing unit normal to the vesicle is $\mathbf{n} = \nabla d$ when computed on Γ. Setting $d^*(\mathbf{x}) = d(\mathbf{x})/\epsilon$, we also require that $\lim_{d^*\to\pm\infty}\phi = \pm 1$ and $\phi = 0$ for $d = 0$. Under this general ansatz, the Ginzburg-Landau free energy $E[\phi]$ recovers the bending and Gaussian terms of the Canham-Helfrich model in the limit of small interface width/vesicle size ratio ($\lambda = \epsilon/D_{ve} \ll 1$). Indeed, a direct substitution of Eq. 9 into Eq. 4 leads to

$$\begin{aligned}E_{\mathrm{B}}[\phi] &= k\frac{3}{4\sqrt{2}}\,\lambda \int_{\overline{\Omega}}\left[\frac{1}{\lambda^2}\left(f'' - \left(f^2 - 1\right)f\right) + \right. \\ &\left. + \frac{1}{\lambda}\left(f'\,\overline{\nabla}\cdot\mathbf{n} + \left(1 - f^2\right)\sqrt{2}\,\overline{m}\right)\right]^2 d\overline{V}\,,\end{aligned} \tag{10}$$

and

$$E_{\mathrm{G}}[\phi] = k_{\mathrm{G}}\frac{35}{16\sqrt{2}}\int_{\overline{\Omega}}\frac{f'^4}{\lambda}\left[\left(\overline{\nabla}\cdot\mathbf{n}\right)^2 + \mathbf{n}\cdot\overline{\nabla}\left(\overline{\nabla}\cdot\mathbf{n}\right)\right]d\overline{V}, \tag{11}$$

where a bar denotes the dimensionless lengths obtained by dividing by D_{ve}. We used the fact that $\nabla\phi(\mathbf{x}) = f'(d^*(\mathbf{x}))\ \mathbf{n}/\epsilon$, where the prime stands for the derivative with respect to $d^*(\mathbf{x})$. A λ expansion of the phase field, $\phi(\mathbf{x}) = f(d^*(\mathbf{x})) = f_0(d^*(\mathbf{x})) + \sum_{i=1}^{+\infty}\lambda^i f_i(d^*(\mathbf{x}))$, shows that $f_0'' = (f_0^2 - 1)f_0$ to minimize the leading order of $E[\phi]$ in the sharp-interface limit $\lambda \ll 1$. This equation admits the solution

$$f_0(d^*(\mathbf{x})) = \tanh\left(\frac{d(\mathbf{x})}{\epsilon\sqrt{2}}\right), \tag{12}$$

which shows that ϵ indeed controls the diffuse interface width. Then, by minimizing the higher-order terms of the expansion, one finds that $f_1(d^*(\mathbf{x})) \equiv 0$ (12). Eventually, since $\sqrt{2}f_0' = (1 - f_0^2)$, the Ginzburg-Landau free energy reads

$$E_{\mathrm{B}}[\phi] = k\frac{3}{4\sqrt{2}}\int_{\overline{\Omega}}\frac{f_0'^2}{\lambda}\left(\overline{\nabla}\cdot\mathbf{n} + 2\overline{m}\right)^2 d\overline{V} + O(\lambda)\,, \tag{13}$$

$$\begin{aligned}E_{\mathrm{G}}[\phi] = k_{\mathrm{G}}\frac{35}{16\sqrt{2}}&\int_{\overline{\Omega}}\frac{f_0'^4}{\lambda}\left[\left(\overline{\nabla}\cdot\mathbf{n}\right)^2\right. \\ &\left. + \mathbf{n}\cdot\overline{\nabla}\left(\overline{\nabla}\cdot\mathbf{n}\right)\right] d\overline{V} + O\left(\lambda^2\right).\end{aligned} \tag{14}$$

If k_1 and k_2 denote the principal curvatures of the ϕ isosurface passing through the generic point $\mathbf{x} \in \Omega$, then $\nabla\cdot\mathbf{n} = -(k_1 + k_2) = -2M(\mathbf{x})$. From differential geometry, $\mathbf{n}\cdot\nabla k_i = k_i^2$, and thus, we also find that $(\nabla\cdot\mathbf{n})^2 + \mathbf{n}\cdot\nabla(\nabla\cdot\mathbf{n}) = 2k_1k_2 = 2G(\mathbf{x})$. Since $\lambda\to 0$, $f_0'^2(\overline{d}(\mathbf{x})/\lambda)/\lambda \overset{w}{\to} 2\sqrt{2}/3\ \delta(\overline{d}(\mathbf{x}))$, and $f_0'^4(\overline{d}(\mathbf{x})/\lambda)/\lambda \overset{w}{\to} 8\sqrt{2}/35\ \delta(\overline{d}(\mathbf{x}))$, where the limits should be understood in a weak sense and $\delta(x)$ is the Dirac delta function, the Ginzburg-Landau free energy recovers the bending and Gaussian components of the Canham-Helfrich free energy

$$E[\phi] \sim 2k\int_{\Gamma}(M - m)^2\, dS + k_{\mathrm{G}}\int_{\Gamma} G dS. \tag{15}$$

Under tension with a nonzero γ, an additional term appears in the Ginzburg-Landau free energy,

$$E[\phi] = E_{\mathrm{T}}[\phi] + E_{\mathrm{B}}[\phi] + E_{\mathrm{G}}[\phi], \tag{16}$$

with

$$E_{\mathrm{T}}[\phi] = \gamma\,\frac{3}{4\sqrt{2}}\,\epsilon\int_{\Omega}\left[\frac{\left(1 - \phi^2\right)^2}{2\epsilon^2} + |\nabla\phi|^2\right]dV. \tag{17}$$

Equation 12 still holds together with $f_1 = 0$ since the integrand in the above equation is subdominant in λ. Therefore,

$$E_{\mathrm{T}}[\phi] = \gamma\, D_{ve}^2\,\frac{3}{2\sqrt{2}}\int_{\overline{\Omega}}\frac{f_0'^2}{\lambda}\, d\overline{V} + O\left(\lambda^2\right), \tag{18}$$

and

$$E_{\mathrm{T}}[\phi] \sim \gamma\int_{\Gamma} dS, \tag{19}$$

in the sharp-interface limit ($\lambda \ll 1$). Since f_0 attains its limiting values ± 1 with an accuracy of about 3% at a distance of $\pm 3\epsilon$ from the $\phi = 0$ bilayer mid-surface, 6ϵ can be matched with the membrane thickness: $6\epsilon = \ell_{me} = 5$ nm (12). Of course, other definitions are possible, but they should be very close to $6\epsilon = \ell_{me}$ since f_0 must just begin to reach its limiting values ± 1 and, e.g., the lateral stress profile must start vanishing. Simulations confirm that the present model recovers the Canham-Helfrich description also in dynamical conditions (12,24). Moreover, the numerical results obtained with the diffuse interface approach are consistent with the experimental evidence, reproducing, e.g., microscopic features such as the stalk-hemifusion intermediates (13,25).

Large spherical vesicles

In what follows, we will calculate the lateral stress profile of large spherical vesicles ($\lambda = \epsilon/D_{ve} \ll 1$). To extract the profile, several integration by

parts of the Ginzburg-Landau free energy must be carried out. Let us initially consider the bending component E_B, which, on the basis of Eq. 13, reads

$$E_B[\phi] = k\frac{3}{\sqrt{2}}\int_\Omega \frac{f_0'^2}{\lambda}(\overline{M} - \overline{m})^2\, d\overline{V} + O(\lambda). \tag{20}$$

For a spherical vesicle, the dimensionless signed distance function reads $d^*(r) = (D_{ve}/2 - r)/\epsilon = (1/2 - \overline{r})/\lambda$. Hence, for a sufficiently large diameter ($\lambda = \epsilon/D_{ve} \ll 1$), we find

$$\begin{aligned} E_B[\phi] &= 4\pi k\frac{3}{\sqrt{2}}\int_0^{+\infty}\frac{f_0'^2\left(\frac{1/2-\overline{r}}{\lambda}\right)}{\lambda}\left(\frac{1}{\overline{r}} - \overline{m}\right)^2 \overline{r}^2 d\,\overline{r} + O(\lambda)\approx \\ &4\pi\int_{-\infty}^{+\infty} z^2\, s_{B2}(z)\, dz + 4\pi D_{ve}\int_{-\infty}^{+\infty} z\, s_{B1}(z)\, dz \\ &+\pi D_{ve}^2\int_{-\infty}^{+\infty} s_{B0}(z)\, dz, \end{aligned} \tag{21}$$

where

$$\begin{aligned} s_{B2}(z) &= \frac{3k}{\sqrt{2}\epsilon^3}\left[f_0''^2(-z/\epsilon) + f_0'(-z/\epsilon)f_0'''(-z/\epsilon)\right], \\ s_{B1}(z) &= -\frac{3}{\sqrt{2}}\frac{2mk}{\epsilon^2}f_0'(-z/\epsilon)f_0''(-z/\epsilon), \\ s_{B0}(z) &= -m^2\frac{3}{\sqrt{2}}\frac{k}{\epsilon}f_0(-z/\epsilon)f_0''(-z/\epsilon), \end{aligned} \tag{22}$$

with $f_0(-z/\epsilon) = \tanh(-z/(\epsilon\sqrt{2}))$ (Eq. 12) and the prime denoting the derivative done with respect to $-z/\epsilon$. Here, $z = r - D_{ve}/2$, so that $z<0$ represents the inner region with the inner leaflet and $z>0$ the outer region with the outer leaflet. See the supporting material for the complete computation. The first integral of the last step of Eq. 21 exactly equals $2k$, the second integral $-2mk$, and the third integral $2km^2$ (see also Eqs. 3a, 3b, and 3c). Overall, the bending energy $E_B[\phi]$ equals $8\pi k - 8\pi mkD_{ve} + 2\pi km^2D_{ve}^2$, which is the Canham-Helfrich bending energy of a sphere. Furthermore, the zeroth and first moments of $s_{B2}(z)$ are zero, the zeroth and second moment of $s_{B1}(z)$ are zero, and the first moment of $s_{B0}(z)$ is zero (its second moment is correctly nonzero and will be discussed later in the section dedicated to the moments of the lateral stress profile). Hence, we can interpret $s_B(z) = s_{B0}(z) + s_{B1}(z) + s_{B2}(z)$ as the bending contribution to the lateral stress profile. By computing the functional derivative of Eq. 4, it is possible to show that $\phi(z) = f_0(-z/\epsilon)$ is the planar solution of the Ginzburg-Landau free energy for $m = 0$. In this case, exploiting the fact that $f_0''' = f_0'(3f_0^2 - 1)$, one can rewrite $s_B(z)$ in the form of Gompper and Zschocke (26), as we explained in our previous work (13). Hence, the result obtained for $s_{B2}(z)$ is exactly that of Gompper and Zschocke, which indeed found a $2k$ contribution to $\mathbb{P}_2^0$. Lázaro et al. (27) calculated the stress tensor associated with $E_B[\phi]$, confirming the result.

Using the same way of reasoning, we calculated the Gaussian contribution to the lateral stress (13). Indeed, starting from Eq. 14, one finds

$$\begin{aligned} E_G[\phi] &= k_G\frac{35}{8\sqrt{2}}4\pi\int_0^{+\infty}\frac{f_0'^4\left(\frac{1/2-\overline{r}}{\lambda}\right)}{\lambda}d\,\overline{r} \\ &+O(\lambda^2) \approx 4\pi\int_{-\infty}^{+\infty} z^2\, s_G(z)dz, \end{aligned} \tag{23}$$

where

$$\begin{aligned} s_G(z) &= k_G\frac{35}{16\sqrt{2}\epsilon^3}\Big[12f_0'^2(-z/\epsilon)f_0''^2(-z/\epsilon) \\ &+ 4f_0'^3(-z/\epsilon)f_0'''(-z/\epsilon)\Big]. \end{aligned} \tag{24}$$

The last integral exactly equals k_G, and therefore, the Gaussian energy equals $4\pi k_G$, which is the Gaussian energy of a sphere in the Canham-Helfrich model. Hence, the second moment of of $s_G(z)$ provides k_G, whereas a direct computation shows that both its zeroth and first moment are zero. Therefore, we interpret $s_G(z)$ as the Gaussian contribution to the lateral stress profile. Eventually,

$$s(z) = s_B(z) + s_G(z) \tag{25}$$

is the lateral stress profile of a membrane vesicle with $\gamma = 0$.

As regards tension, from Eq. 18, one finds that

$$\begin{aligned} E_T[\phi] &= \gamma D_{ve}^2\frac{3}{2\sqrt{2}}4\pi\int_0^{+\infty}\frac{f_0'^2\left(\frac{1/2-\overline{r}}{\lambda}\right)}{\lambda}\overline{r}^2 d\,\overline{r} \\ &+O(\lambda^2)\approx \pi D_{ve}^2\int_{-\infty}^{+\infty} s_T(z)dz. \end{aligned} \tag{26}$$

The last integral exactly equals γ. Furthermore, the first moment of $s_T(z)$ is zero, whereas the second moment is correctly nonzero and will be discussed later. Therefore, we interpret

$$s_T(z) = -\gamma\frac{3}{2\sqrt{2}}\frac{1}{\epsilon}f_0(-z/\epsilon)f_0''(-z/\epsilon) \tag{27}$$

as the γ contribution to the lateral stress profile. All the integration by parts performed in the integrals above to obtain the lateral stress contributions are explicitly reported in the supporting material.

RESULTS

Bending contribution to the lateral stress

The diffuse interface approach replaces the sharp interface of the Canham-Helfrich model with a diffuse layer, preserving its elasticity. This makes it possible to take into account the scale of the membrane thickness, which is directly related to the thickness of the diffuse interface, in turn controlled by the small parameter ϵ. As explained in the materials and methods, we set $6\epsilon = 5$ nm. The bending contribution to the lateral stress of a large spherical vesicle is

$$s_B(z) = s_{B2}(z) + s_{B1}(z) + s_{B0}(z), \tag{28}$$

where

$$s_{B2}(z) = \frac{3k}{\sqrt{2}\epsilon^3}\left[f_0''^2(-z/\epsilon) + f_0'(-z/\epsilon)f_0'''(-z/\epsilon)\right],$$

$$s_{B1}(z) = -\frac{3}{\sqrt{2}}\frac{2mk}{\epsilon^2}f_0'(-z/\epsilon)f_0''(-z/\epsilon),$$

$$s_{B0}(z) = -m^2\frac{3}{\sqrt{2}}\frac{k}{\epsilon}f_0(-z/\epsilon)f_0''(-z/\epsilon), \tag{29}$$

with $f_0(-z/\epsilon) = \tanh(-z/(\epsilon\sqrt{2}))$, and the prime denoting the derivative done with respect to $-z/\epsilon$. Here, $z < 0$ represents the inner region with the inner leaflet and $z > 0$ the outer region with the outer leaflet. Hence, the bending contribution to the lateral stress is the superposition of three terms. A direct calculation shows that the second moment of $s_{B2}(z)$ exactly equals $2k$, whereas its first and zeroth moments are zero. Indeed, $s_{B2}(z)$ is a zero mean, even function, directly proportional to k and independent of m. $s_{B1}(z)$ contributes only to the first moment, as it is an odd function and thus uniquely provides a tension difference between the two monolayers, which vanishes for $m = 0$ (28,29). This is mechanically correct since the first moment is nothing but a bending torque, and therefore, a couple should be present. Hence, it provides a zero net bilayer tension. The first moment of $s_{B1}(z)$ turns out to be $-2km$. The even function $s_{B0}(z)$, proportional to m^2, yields the spontaneous tension through its zeroth moment and also contributes to the second moment of the lateral stress, as will be discussed later. Fig. 1 shows the three terms of the bending contribution to the lateral stress. Of course, for a symmetric membrane, $s_{B1}(z) = s_{B0}(z) = 0$ since $m = 0$.

Gaussian contribution to the lateral stress

The Gaussian contribution to the lateral stress is

$$s_G(z) = k_G\frac{35}{16\sqrt{2}\epsilon^3}\left[12f_0'^2(-z/\epsilon)f_0''^2(-z/\epsilon) + 4f_0'^3(-z/\epsilon)f_0'''(-z/\epsilon)\right], \tag{30}$$

which is a zero mean, even function (see Fig. 2). Therefore, the zeroth and first moments of $s_G(z)$ are zero. The second moment is instead equal to k_G. Therefore,

$$s(z) = s_B(z) + s_G(z) \tag{31}$$

is the lateral stress profile of a membrane vesicle with $\gamma = 0$. Fig. 3 depicts the lateral stress profile $s(z) = s_B(z) + s_G(z)$. The plot assumes $k = 20\ k_BT$, but it can be directly rescaled for any other value of k. The Gaussian modulus is $k_G = -0.7k$, and the black line with circles shows $s(z)$ for a symmetric membrane, namely with $m = 0$. The profile is attractive (positive) in the regions of the headgroups, which therefore tend to minimize the contact area with the surrounding aqueous environments. The profile is repulsive (negative) inside the bilayer, namely in the lipid tail region. At the bilayer mid-surface, a mildly positive stress bump is present, denoting a small surface tension between the two monolayers. The height of this central peak is controlled by the ratio k_G/k, as shown in Fig. 4. We extensively discussed this behavior in our previous work (13), and we will further discuss it here. Anyway, here, we focus on the case $k_G = -0.7k$, which is the value found by Hu et al. (5) with the MARTINI model if one considers an updated value for the bending rigidity (15). The consequent small central bump is also observed in molecular simulations (30–32) (see also the *inset* of Fig. 3). One may notice that, in Fig. 3, the lateral stress profile is plotted for $k = 20\ k_BT$. Therefore, the precise height of the peaks can be modulated simply by changing this value, which, in many

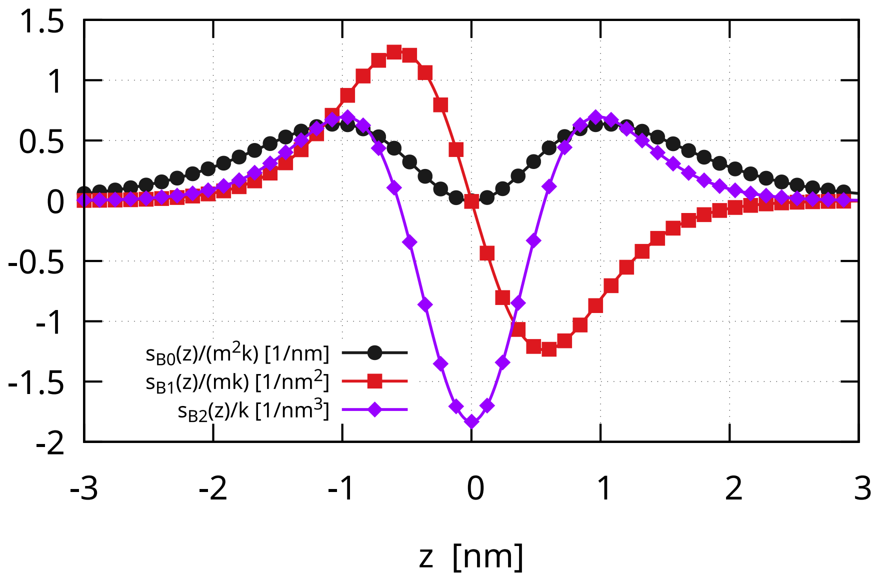

FIGURE 1 The three terms of the bending contribution to the lateral stress profile, $s_B(z) = s_{B2}(z) + s_{B1}(z) + s_{B0}(z)$. Each term is normalized so that it does not depend on the elastic constants. $s_{B2}(z)$ only depends upon k and is a zero mean, even function. $s_{B1}(z)$ is an odd function, which provides a tension difference between the two leaflets but a zero net bilayer tension. The sign of this difference depends on the sign of m, providing a bending moment that balances the tendency of the membrane segment to bend toward the exterior ($m > 0$) or toward the interior ($m < 0$). $s_{B0}(z)$ always provides a nonnegative tension to the two leaflets and therefore to the bilayer. Of course, for a symmetric membrane, $s_{B1}(z)$ and $s_{B0}(z)$ vanish since $m = 0$. Here, $6\epsilon = 5$ nm, $z > 0$ represents the outer leaflet and $z < 0$ the inner leaflet.

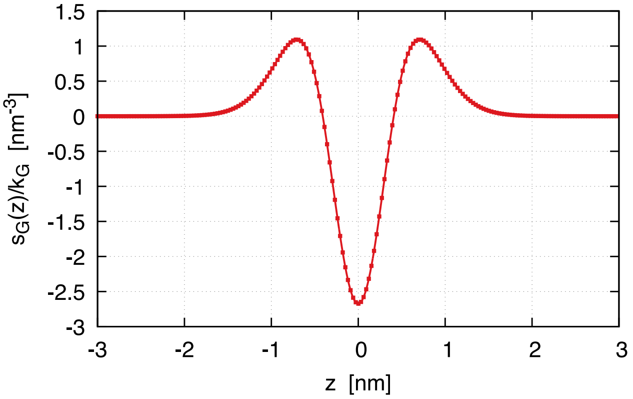

FIGURE 2 The Gaussian contribution $s_G(z)$ to the lateral stress profile. The curve is normalized with the Gaussian modulus k_G. $s_G(z)$ is a zero mean, even function, so that it only contributes to the second moment of the lateral stress. The shape of $s_G(z)/k_G$ is similar to that of $s_{B2}(z)/k$ but is actually different. More importantly, due to stability arguments, k_G should be negative, and therefore, $s_G(z)$ is actually reversed. Here, $6\epsilon = 5$ nm, $z>0$ represents the outer leaflet and $z<0$ the inner leaflet.

cases, can easily be twice as much (2). The case of asymmetric membranes is depicted in Fig. 3. The red line with squares shows the case of a positive bilayer spontaneous curvature, $m = 1/10\ \mathrm{nm}^{-1}$. A $m>0$ means that the membrane would like to bend toward the exterior ($z>0$ side). Hence, in the given equilibrium, the inner monolayer is more attractive, and therefore, the stress profile is more positive in its region, whereas the outer monolayer is more repulsive, and therefore, the stress profile is more negative. The same trend is apparent in the profiles obtained by Ghosh et al. (33) with dissipative particle dynamics, where a positive m was induced by the membrane adsorption of small solutes. The blue line with diamonds in Fig. 3 shows the case of a negative bilayer spontaneous curvature, $m = -1/10\ \mathrm{nm}^{-1}$, where the behavior is the opposite of the one just described. Overall, the profile we obtain is a coarse-grained version of those found in molecular simulations (4,5,8,30–32,34), with peaks that have the same order of magnitude (hundreds of bars).

The γ contribution

The γ contribution to the lateral stress profile is

$$s_T(z) = -\gamma \frac{3}{2\sqrt{2}} \frac{1}{\epsilon} f_0(-z/\epsilon) f_0''(-z/\epsilon), \qquad (32)$$

which is an even function. The zeroth moment of $s_T(z)$ exactly equals γ, whereas the first moment is zero. The second moment is correctly nonzero and will be discussed later. One may notice that $s_T(z)/\gamma = s_{B0}(z)/(2km^2)$, which is in accordance with the fact that both contribute to the total tension $\Sigma = \gamma + \widehat{\Sigma}$, where $\widehat{\Sigma} = 2km^2$ is the spontaneous tension. Fig. 5 *a* shows the contribution provided by $s_T(z)$ to the lateral stress for three typical values of γ (35): $\gamma = 0.01$ mN/m (*blue line with diamonds*), $\gamma = 0.02$ mN/m (*black line with circles*), and $\gamma = 0.04$ mN/m (*red line with squares*). Therefore, $s_T(z)$ has a structure that provides two surface tensions to the bilayer, leading to two stretched leaflets. Of course, a negative γ would reverse the plots, leading to two compressed leaflets. Anyway, given the small values of γ typical of fluid lipid bilayers, $s_T(z)$ does not contribute significantly to the lateral stress profile and, moreover, does not provide tension differences between the two monolayers. However, when a bilayer is stretched (compressed), the membrane thins (thickens). Fig. 5 *b* shows the total lateral stress profile, $s(z) = s_T(z) + s_B(z) + s_G(z)$, of a symmetric membrane ($k = 20\ k_BT$, $k_G = -0.7k$, $m = 0$) for three different diffuse interface widths: $6\epsilon = 4.5$ nm (*green line with squares*), $6\epsilon = 4.75$ nm (*orange line with circles*), and the standard $6\epsilon = 5$ nm (*purple line with diamonds*). As is evident, when the membrane thins, the peak-to-peak distance

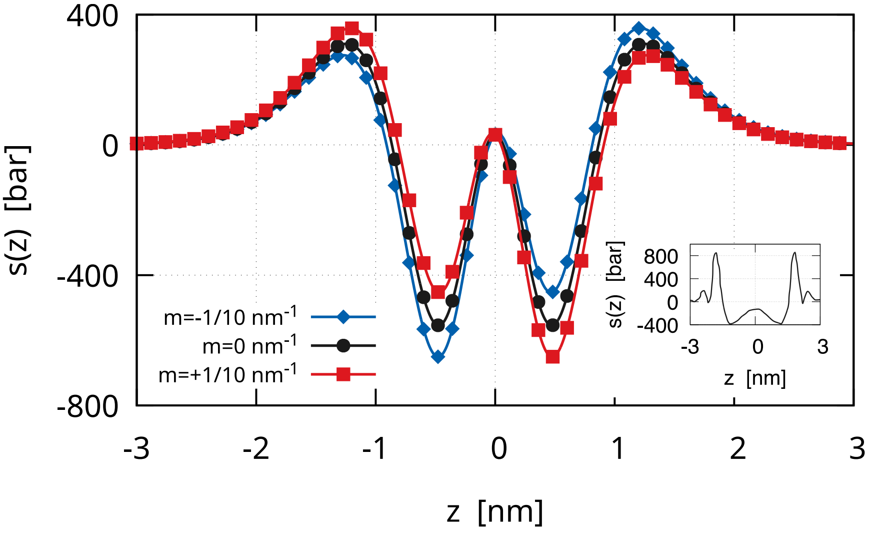

FIGURE 3 The lateral stress profile $s(z) = s_B(z) + s_G(z)$ due to the bending and Gaussian energy contributions of a spherical membrane vesicle as revealed by the diffuse interface approach. The plot assumes $k = 20\ k_BT$, but it can be directly rescaled for any other value of k. The Gaussian modulus is $k_G = -0.7k$. The black line with circles shows $s(z)$ for a symmetric membrane ($m = 0$) so that $s_{B0}(z) = s_{B1}(z) = 0$. The red line with squares depicts the case of an asymmetric membrane with $m = +1/10\ \mathrm{nm}^{-1}$. The blue line with diamonds shows the case with $m = -1/10\ \mathrm{nm}^{-1}$. Here, $6\epsilon = 5$ nm, $z>0$ represents the outer leaflet and $z<0$ the inner leaflet. The inset sketches the lateral stress profile of a symmetric POPE membrane obtained with a coarse-grained molecular dynamics simulation in (30). Different profiles can be obtained with different molecular models, as we reviewed in (13).

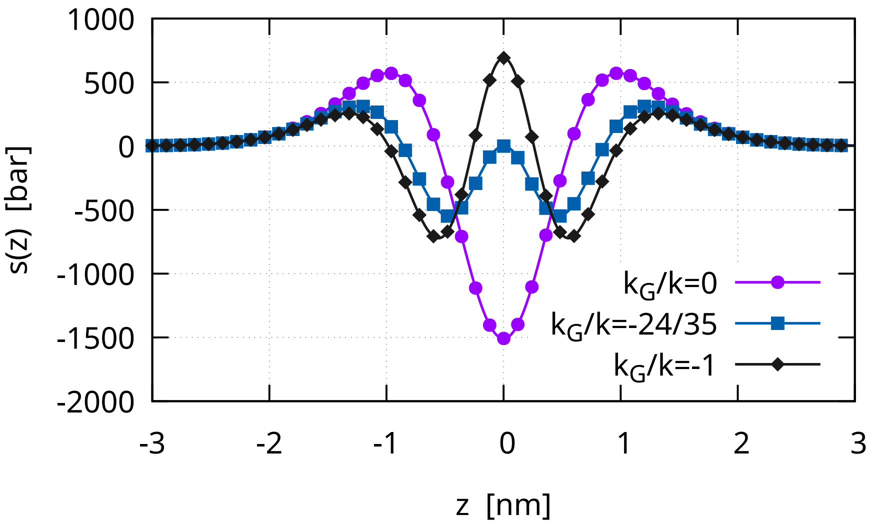

FIGURE 4 The lateral stress profile $s(z) = s_B(z) + s_G(z)$ of a symmetric large spherical vesicle ($m = 0$), with $k = 20\ k_BT$. The ratio k_G/k controls the appearance of a central stress bump, whose presence is balanced by the decrease of the positive peaks in the headgroup regions since the membrane is tensionless. When $k_G/k = -24/35$, the central peak is exactly zero. Here, $6\epsilon = 5$ nm, $z > 0$ represents the outer leaflet and $z < 0$ the inner leaflet.

decreases, and the absolute value of both the positive and negative peaks increases. This is also the behavior Ting and Müller found with self-consistent field theory for membranes under tension (31).

Moments of the lateral stress

Now, we evaluate the moments of the lateral stress profile $s(z) = s_T(z) + s_B(z) + s_G(z)$, where $s_T(z)$ is given by Eq. 27, $s_B(z)$ by Eq. 28, and $s_G(z)$ by Eq. 24. As regards this last contribution, in what follows, we make the change $k_G \to k_G|_{\Sigma=0}$ since it concerns the intrinsic Gaussian modulus, therefore without tension. In other words, the Gaussian contribution to the lateral stress profile reads

$$s_G(z) = k_G|_{\Sigma=0} \frac{35}{16\sqrt{2}\epsilon^3}\Big[12 f_0'^2(-z/\epsilon) f_0''^2(-z/\epsilon) + 4 f_0'^3(-z/\epsilon) f_0'''(-z/\epsilon)\Big], \quad (33)$$

where the only difference with Eq. 24 is the notation for the Gaussian modulus. This change is needed since it is known that tension affects the Gaussian modulus, as follows from the monolayer-bilayer consistency relations. In fact, it is possible to match the Canham-Helfrich elastic constants of the bilayer with those of the constituent monolayers (14,36):

$$\Sigma = \Sigma_{m1} + \Sigma_{m2}, \quad (34a)$$

$$k = k_{m1} + k_{m2}, \quad (34b)$$

$$m = \frac{k_{m1} m_{m1} - k_{m2} m_{m2}}{k_{m1} + k_{m2}} + z_0 \frac{\Sigma_{m1} - \Sigma_{m2}}{2(k_{m1} + k_{m2})}, \text{ and} \quad (34c)$$

$$k_G = k_{Gm1} + k_{Gm2} - 4 z_0 (k_{m1} m_{m1} + k_{m2} m_{m2}) + z_0^2 \Sigma. \quad (34d)$$

Here, Σ_{mi} are the tensions, k_{mi} the bending rigidities, m_{mi} the spontaneous curvatures, and k_{Gmi} the Gaussian moduli (which is usually negative (36)) of the two monolayers. In Eqs. 34c and 34d, the monolayers are assumed to have two neutral surfaces—the surfaces at which bending and stretching are decoupled—located at $z_{m1} = -z_{m2} = z_0 > 0$ with respect to the bilayer mid-surface. Usually, in Eq. 34c, the first-order correction in z_0 is neglected, and one may notice that, in the case of a symmetric membrane, m vanishes since $k_{m1} = k_{m2}$ and $m_{m1} = m_{m2}$ (and $\Sigma_{m1} = \Sigma_{m2}$). Even in the case of symmetric membranes, the monolayer spontaneous curvature affects the bilayer Gaussian modulus k_G (Eq. 34d), which also depends on the bilayer tension Σ, albeit at second order in z_0 (5). Hence, one can write $k_G = k_G|_{\Sigma=0} + z_0^2\Sigma$.

A direct computation of the moments of the lateral stress profile of the diffuse interface leads to

$$\int_{-\infty}^{+\infty} s(z)dz = \gamma + 2km^2 = \Sigma, \quad (35a)$$

$$\int_{-\infty}^{+\infty} z s(z)dz = -2km, \quad (35b)$$

$$\int_{-\infty}^{+\infty} z^2 s(z)dz = 2k + k_G|_{\Sigma=0} + \frac{12+\pi^2}{54}\left(\frac{\ell_{me}}{2}\right)^2 \Sigma. \quad (35c)$$

These results should be interpreted as $\mathbb{P}_0^0$, $\mathbb{P}_1^0$, and $\mathbb{P}_2^0$. In the second moment, $\ell_{me} = 6\epsilon$ is the membrane thickness, and a $2k$ contribution was also found by Gompper and Zschocke (26), and by Lázaro et al. (27) through a direct calculation of the stress tensor. It is thermodynamically consistent (Eq. 3c). The elastic constants controlling the lateral stress profile we calculated are compatible with those at constant chemical potential, but our results were obtained

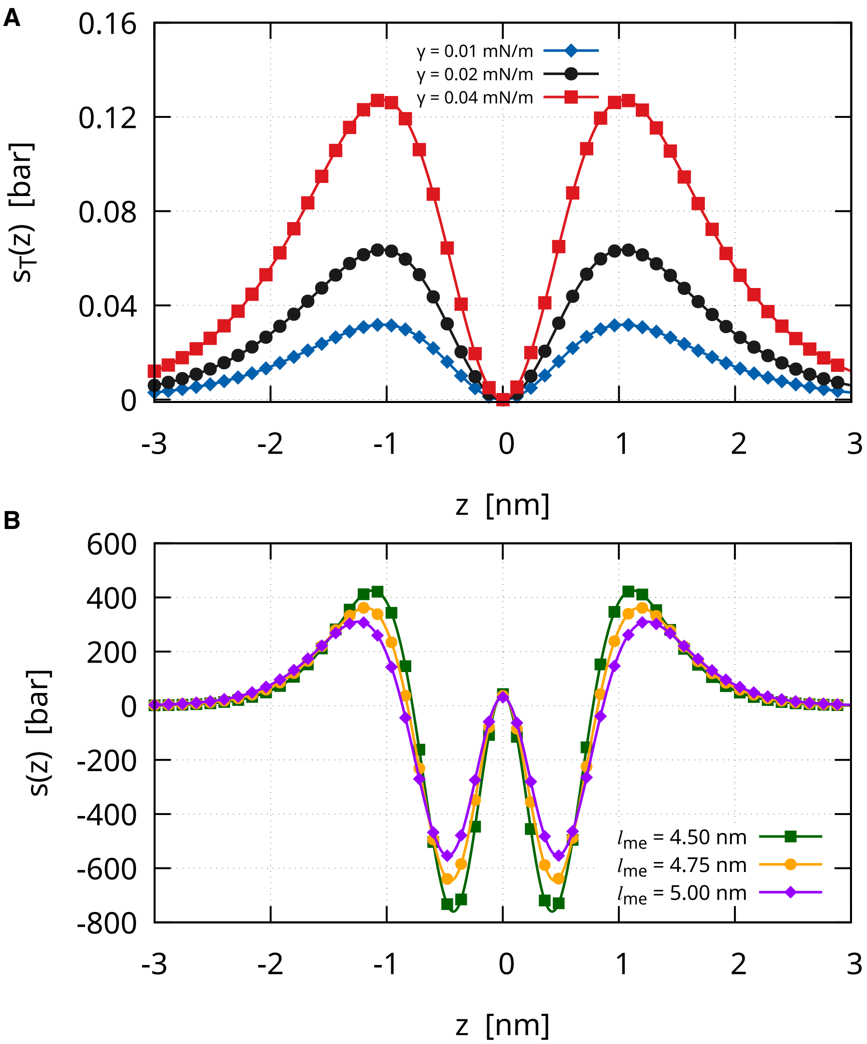

FIGURE 5 (*a*) The γ contribution to the lateral stress profile $s_{\mathrm{T}}(z)$ for three different tensions: $\gamma = 0.01$ mN/m (*blue line with diamonds*), $\gamma = 0.02$ mN/m (*black line with circles*), and $\gamma = 0.04$ mN/m (*red line with squares*). Of course, negative values of γ reverse the plot. (*b*) The total lateral stress profile of the diffuse interface ($k = 20\ k_B T$, $k_{\mathrm{G}} = -0.7k$, $m = 0$), $s(z) = s_{\mathrm{T}}(z) + s_{\mathrm{B}}(z) + s_{\mathrm{G}}(z)$, for three different membrane thicknesses: $6\epsilon = 4.5$ nm (*green line with squares*), $6\epsilon = 4.75$ nm (*orange line with circles*), and the standard $6\epsilon = 5$ nm (*purple line with diamonds*).

independently of Eqs. 3a, 3b, and 3c (see also the remarks given in Lázaro et al. (27)). Notice that, in our former works (12,13) on topological transitions of lipid vesicles, γ was understood as a Lagrange multiplier to enforce surface area conservation, and we also constrained the enclosed volume of vesicles.

The second moment can be rewritten as

$$\int_{-\infty}^{+\infty} z^2 s(z) dz = 2k + k_{\mathrm{G}}|_{\Sigma=0} + {z_D}^2 \Sigma, \tag{36}$$

setting

$$z_D^2 = \frac{12+\pi^2}{54}\left(\frac{\ell_{me}}{2}\right)^2, \tag{37}$$

which provides a tension correction, with the same structure of that in the monolayer-bilayer consistency relation (Eq. 34d). Astonishingly,

$$z_D = \sqrt{\frac{12+\pi^2}{54}}\left(\frac{\ell_{me}}{2}\right) \approx 0.64\left(\frac{\ell_{me}}{2}\right), \tag{38}$$

which matches the expected value of z_0, according to the rule of thumb that places the monolayer neutral plane at about 2/3 of the length of a lipid (10,15). One may notice that, in our previous work (13), the $z_D^2\Sigma$ term was neglected since it makes a small correction that vanishes with $\lambda^2 = \epsilon^2/D_{ve}^2$—it is due to a finite thickness of the interface and therefore vanishes in the sharp-interface limit. One would find that $z_D = (2/3)(\ell_{me}/2)$, setting $\ell_{me} = 5.73\epsilon$, which corresponds to defining an interface width slightly smaller than the 6ϵ we usually consider (12,13,24,25). On the one hand, such a tension correction is not entirely surprising given that analogs of the monolayer-bilayer consistency relations can be obtained through combinations of the moments of the monolayers and of the bilayer, as reported e.g., in Hu et al. (5). On the contrary, what was hard to expect, and not easy to explain, is the numerical value of

z_D, which matches the expected value of z_0. The actual location of the neutral plane should be addressed by means of the local stretching modulus distribution, as explained in Campelo et al. (37). Anyway, given the coarse-grained nature and aims of the diffuse interface model, we believe the dependence of $\mathbb{P}_2^0$ on $z_D^2\Sigma$ is a pleasant result.

DISCUSSION

Besides being used in molecular simulations to determine elastic constants, the lateral stress profile has important implications for membrane-protein interactions (38,39). For example, Gullingsrud and Shulten (40) estimated the work required for gating a mechanosensitive channel MscL by measuring the membrane lateral stress profile. This work that the protein has to perform against the bilayer lateral pressure contributes significantly to the gating (41,42) but can be lowered if the membrane is asymmetric, namely if there is a nonvanishing bilayer spontaneous curvature. This role of m is remarkable since the plasma membrane is asymmetric. Furthermore, it has recently been shown experimentally that an asymmetric lateral stress profile can serve as the dominant factor in controlling membrane-protein activity (43) (see also Martinac et al. (44)). Interestingly, the lateral stress distribution can be made asymmetric by the unilateral insertion of various amphiphatic molecules, like antipsychotic drugs or local anesthetics (44)—on the basis of Eq. 34c, a molecule that generates a positive monolayer spontaneous curvature produces a positive m if inserted in the outer leaflet and a negative m if inserted in the inner leaflet. These insertions might not only be related to the generation of a bilayer spontaneous curvature m but also to a change in the Gaussian modulus k_G. Indeed, a molecule that preferentially inserts into the lipid head region generates a positive monolayer spontaneous curvature, which, on the basis of Eq. 34d, tends to make k_G even more negative. This effect on k_G has also been pointed out by Downing et al. (45) with a molecular lipid model. A more negative k_G is the opposite of what is desired for enhancing membrane fusion, which should be facilitated by a deep insertion in the hydrocarbon chain region to render k_G less negative (through the generation of negative monolayer spontaneous curvature, Eq. 34c). A change in k_G/k alters the shape of the lateral stress profile (Fig. 4). As we discussed in our previous work (13), the diffuse interface captures a change of the profile with k_G/k, which is consistent with shallow/deep insertions of molecules within the bilayer. For example, Fig. 6 (*black line with circles*) shows the lateral stress profile for $k_G/k = -0.8$ for a symmetric ($m = 0$), tensionless membrane. This case corresponds to that of a symmetric, shallow insertion of molecules within the bilayer, which coherently results in a mitigation of the attraction between lipid heads compared to the case $k_G/k = -0.7$ depicted in Fig. 3. Since the membrane is tensionless, this reduction is balanced by a more pronounced central stress bump. If the insertion is unilateral, then one is left with a nonzero bilayer spontaneous curvature, which leads to an asymmetric profile with positive peaks more mitigated on the side of the shallow insertion (*red line with squares* and *blue line with diamonds* in Fig. 6). In fact, the shallow insertion of molecules in the outer leaflet produces a positive m. The same effect might be obtained by the deep insertion of molecules in the internal leaflet, which produces a $m > 0$ through the generation of negative (inner) monoalyer spontaneous curvature, even if, in this case, k_G would increase. Similarly, the deep insertion in the outer leaflet produces a $m < 0$ as well as an increase of k_G, whereas the shallow insertion in the inner leaflet leads to a $m < 0$ with a decrease of k_G. Finally, it is worth saying that the Σ correction to the Gaussian modulus is usually negligible given the small tensions that bilayer membranes can sustain. This correction might only be important when a dynamic tension is applied, which can be several order of magnitude larger (46).

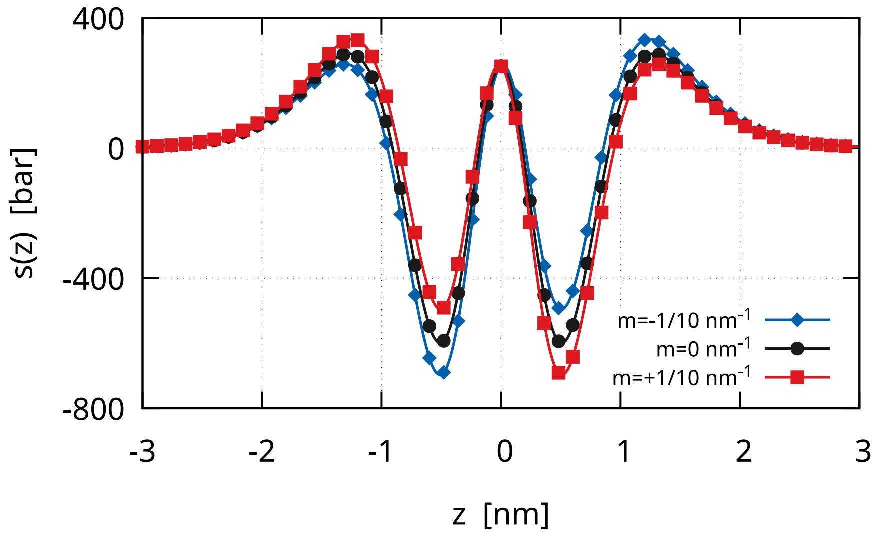

FIGURE 6 The lateral stress profiles $s(z)$ of a membrane with $k = 20\ k_BT$, $k_G = -0.8k$, and $\gamma = 0$. The black line with circles shows $s(z)$ for a symmetric membrane ($m = 0$), which is therefore tensionless. The red line with squares depicts the case in which the membrane is asymmetric, with $m = +1/10\ \text{nm}^{-1}$. The blue line with diamonds shows the case with $m = -1/10\ \text{nm}^{-1}$.

Our results show the general behavior of the lateral stress with respect to the insertion of molecules within the membrane. This behavior is captured by means of few macroscopic elastic constants, which can be observed and controlled. Interestingly, distinct local anesthetics insert differently within the membrane, and the coarse-grained picture we obtain might be useful to explain the multitude of target proteins for the same molecule as well as the fact that various anesthetics can act on the same target protein: this might happen through the modification of the lateral stress profile (47), which influences part of the work required to gate channels (38,40).

CONCLUSION

In this work, we have analyzed the lateral stress profile provided by the diffuse interface approach, which has the unique feature to be an analytical function of the Canham-Helfrich elastic constants. We showed that also higher-order corrections to the second moment are captured, providing a tension-dependent, second-order correction in the membrane thickness (z_D). In particular, the model provides a value for z_D that is $2/3$ of the length of a monolayer. This value coincides numerically with the expected location of the monolayers' neutral surfaces. These findings, together with the general result that the lateral stress profile we obtain is a coarse-grained version of those found with molecular models, shows that the approach is much richer in detail than one might think based only on the sharp-interface limit to the Canham-Helfrich elastic energy. In fact, the presence of such details is indeed compatible with the derivation of the Ginzburg-Landau free energy since elasticity is nothing but the emergence at a larger scale of the amphiphilic structure of the bilayer. We also discussed the results with respect to the insertion of molecules, which play a crucial role in modifying membrane-protein interactions. Besides this, our results further corroborate the fact that the diffuse interface allows simulations that simultaneously contain the large scale of vesicles and the small scale of the bilayer thickness (13).

ACKNOWLEDGMENTS

The research has received financial support from ICSC-Italian Research Center on High Performance Computing, Big Data, and Quantum Computing, funded by European Union-NextGenerationEU. Support is acknowledged from the 2024 Sapienza Large Project RG124190FE08089A and from the 2024 Sapienza Avvio alla ricerca project AR2241907893A818. Concerning computational resources, we acknowledge the CINECA award under the ISCRA initiative for making available high-performance computing resources and providing support (ISCRA-B D-RESIN, ISCRA-B CAMAGE3D, and ISCRA-C GaVesFu).

AUTHOR CONTRIBUTIONS

C.M.C. designed the study; M. Bottacchiari carried out the lateral stress profile calculations and analyzed data with M. Bussoletti under the supervision of M.G.; and M. Bottacchiari wrote the paper with contributions from all authors. All authors discussed the results and read, revised, and approved the final version.

DECLARATION OF INTERESTS

The authors declare no competing interests.

SUPPORTING MATERIAL

Supporting material can be found online at https://doi.org/10.1016/j.bpj.2025.07.041.

REFERENCES

1. Dimova, R. 2014. Recent developments in the field of bending rigidity measurements on membranes. *Adv. Colloid Interface Sci.* 208:225–234.
2. Karal, M. A. S., M. M. Billah, …, M. K. Ahamed. 2023. A review on the measurement of bending rigidity of lipid membranes. *Soft Matter.* 19:8285–8304.
3. Steinkühler, J., R. L. Knorr, …, R. Lipowsky. 2020. Controlled division of cell-sized vesicles by low densities of membrane-bound proteins. *Nat. Commun.* 11:905.
4. Hu, M., J. J. Briguglio, and M. Deserno. 2012. Determining the Gaussian curvature modulus of lipid membranes in simulations. *Biophys. J.* 102:1403–1410.
5. Hu, M., D. H. de Jong, …, M. Deserno. 2013. Gaussian curvature elasticity determined from global shape transformations and local stress distributions: a comparative study using the MARTINI model. *Faraday Discuss.* 161:365–459.
6. Oversteegen, S. M., and F. Leermakers. 2000. Thermodynamics and mechanics of bilayer membranes. *Phys. Rev.* 62:8453–8461.
7. Martinus Oversteegen, S., P. A. Barneveld, …, J. Lyklema. 1999. Thermodynamic derivation of mechanical expressions for interfacial parameters. *Phys. Chem. Phys.* 1:4987–4994.
8. Nakagawa, K. M., and H. Noguchi. 2016. Nonuniqueness of local stress of three-body potentials in molecular simulations. *Phys. Rev.* 94:053304.
9. Lipowsky, R. 2014. Coupling of bending and stretching deformations in vesicle membranes. *Adv. Colloid Interface Sci.* 208:14–24.
10. Kozlov, M. M., and M. Winterhalter. 1991. Elastic moduli for strongly curved monoplayers. Position of the neutral surface. *J. Phys. II France.* 1:1077–1084.
11. Hu, T., H. Wang, and H. Gomez. 2023. Direct van der Waals simulation (DVS) of phase-transforming fluids. *Sci. Adv.* 9:eadg3007.
12. Bottacchiari, M., M. Gallo, …, C. M. Casciola. 2022. Activation energy and force fields during topological transitions of fluid lipid vesicles. *Commun. Phys.* 5:283.
13. Bottacchiari, M., M. Gallo, …, C. M. Casciola. 2024. The diffuse interface description of fluid lipid membranes captures key features of the hemifusion pathway and lateral stress profile. *PNAS Nexus.* 3:pgae300.
14. Deserno, M. 2015. Fluid lipid membranes: From differential geometry to curvature stresses. *Chem. Phys. Lipids.* 185:11–45.
15. Wang, X., and M. Deserno. 2015. Determining the pivotal plane of fluid lipid membranes in simulations. *J. Chem. Phys.* 143:164109.
16. Du, Q., C. Liu, and X. Wang. 2004. A phase field approach in the numerical study of the elastic bending energy for vesicle membranes. *J. Comput. Phys.* 198:450–468.
17. Du, Q., C. Liu, …, X. Wang. 2005. Modeling the spontaneous curvature effects in static cell membrane deformations by a phase field formulation. *Commun. Pure Appl. Anal.* 4:537–548.

18. Du, Q., C. Liu, and X. Wang. 2006. Simulating the deformation of vesicle membranes under elastic bending energy in three dimensions. *J. Comput. Phys.* 212:757–777.

19. Campelo, F., and A. Hernández-Machado. 2006. Dynamic model and stationary shapes of fluid vesicles. *Eur. Phys. J. E.* 20:37–45.

20. Campelo, F., and A. Hernández-Machado. 2007. Model for curvature-driven pearling instability in membranes. *Phys. Rev. Lett.* 99:088101.

21. Gu, R., X. Wang, and M. Gunzburger. 2014. Simulating vesicle-substrate adhesion using two phase field functions. *J. Comput. Phys.* 275:626–641.

22. Lázaro, G., A. Hernández-Machado, and I. Pagonabarraga. 2014. Rheology of red blood cells under flow in highly confined microchannels: I. effect of elasticity. *Soft Matter.* 10:7195–7206.

23. Kusumaatmaja, H. 2015. Surveying the free energy landscapes of continuum models: Application to soft matter systems. *J. Chem. Phys.* 142:12.

24. Bussoletti, M., M. Gallo, …, C. M. Casciola. 2024. Mesoscopic elasticity controls dynamin-driven fission of lipid tubules. *Sci. Rep.* 14:14003.

25. Bottacchiari, M., M. Gallo, …, C. M. Casciola. 2024. The local variation of the Gaussian modulus enables different pathways for fluid lipid vesicle fusion. *Sci. Rep.* 14:23.

26. Gompper, G., and S. Zschocke. 1992. Ginzburg-Landau theory of oil-water-surfactant mixtures. *Phys. Rev. A.* 46:4836–4851.

27. Lázaro, G. R., I. Pagonabarraga, and A. Hernández-Machado. 2015. Phase-field theories for mathematical modeling of biological membranes. *Chem. Phys. Lipids.* 185:46–60.

28. Miettinen, M. S., and R. Lipowsky. 2019. Bilayer membranes with frequent flip-flops have tensionless leaflets. *Nano Lett.* 19:5011–5016.

29. Hossein, A., and M. Deserno. 2020. Spontaneous curvature, differential stress, and bending modulus of asymmetric lipid membranes. *Biophys. J.* 118:624–642.

30. Vanegas, J. M., A. Torres-Sánchez, and M. Arroyo. 2014. Importance of force decomposition for local stress calculations in biomembrane molecular simulations. *J. Chem. Theory Comput.* 10:691–702.

31. Ting, C. L., and M. Müller. 2017. Membrane stress profiles from self-consistent field theory. *J. Chem. Phys.* 146:10.

32. Zoni, V., P. Campomanes, and S. Vanni. 2021. Investigating the structural properties of hydrophobic solvent-rich lipid bilayers. *Soft Matter.* 17:5329–5335.

33. Ghosh, R., V. Satarifard, …, R. Lipowsky. 2021. Budding and Fission of Nanovesicles Induced by Membrane Adsorption of Small Solutes. *ACS Nano.* 15:7237–7248.

34. Różycki, B., and R. Lipowsky. 2015. Spontaneous curvature of bilayer membranes from molecular simulations: Asymmetric lipid densities and asymmetric adsorption. *J. Chem. Phys.* 142:054101.

35. Kozlov, M. M., and L. V. Chernomordik. 2015. Membrane tension and membrane fusion. *Curr. Opin. Struct. Biol.* 33:61–67.

36. Siegel, D. P., and M. M. Kozlov. 2004. The gaussian curvature elastic modulus of N-monomethylated dioleoylphosphatidylethanolamine: relevance to membrane fusion and lipid phase behavior. *Biophys. J.* 87:366–374.

37. Campelo, F., C. Arnarez, …, M. M. Kozlov. 2014. Helfrich model of membrane bending: from Gibbs theory of liquid interfaces to membranes as thick anisotropic elastic layers. *Adv. Colloid Interface Sci.* 208:25–33.

38. Cantor, R. S. 1997. Lateral pressures in cell membranes: a mechanism for modulation of protein function. *J. Phys. Chem. B.* 101:1723–1725.

39. Marsh, D. 2007. Lateral pressure profile, spontaneous curvature frustration, and the incorporation and conformation of proteins in membranes. *Biophys. J.* 93:3884–3899.

40. Gullingsrud, J., and K. Schulten. 2004. Lipid bilayer pressure profiles and mechanosensitive channel gating. *Biophys. J.* 86:3496–3509.

41. Sukharev, S. I., W. J. Sigurdson, …, F. Sachs. 1999. Energetic and spatial parameters for gating of the bacterial large conductance mechanosensitive channel, MscL. *J. Gen. Physiol.* 113:525–540.

42. Samuli Ollila, O. H., T. Róg, …, I. Vattulainen. 2007. Role of sterol type on lateral pressure profiles of lipid membranes affecting membrane protein functionality: comparison between cholesterol, desmosterol, 7-dehydrocholesterol and ketosterol. *J. Struct. Biol.* 159:311–323.

43. Piller, P., E. F. Semeraro, …, G. Pabst. 2023. Allosteric modulation of integral protein activity by differential stress in asymmetric membranes. *PNAS Nexus.* 2:pgad126.

44. Martinac, B., N. Bavi, …, O. Bavi. 2018. Tuning ion channel mechanosensitivity by asymmetry of the transbilayer pressure profile. *Biophys. Rev.* 10:1377–1384.

45. Downing, R., G. Volpe Bossa, and S. May. 2020. Saddle-curvature instability of lipid bilayer induced by amphipathic peptides: A molecular model. *Soft Matter.* 16:5032–5043.

46. Evans, E., V. Heinrich, …, W. Rawicz. 2003. Dynamic tension spectroscopy and strength of biomembranes. *Biophys. J.* 85:2342–2350.

47. Grage, S. L., A. Culetto, …, S. Weinschenk. 2021. Membrane-mediated activity of local anesthetics. *Mol. Pharmacol.* 100:502–512.

Biophysical Journal, Volume *124*

Supplemental information

The lateral stress profile of fluid lipid membranes as revealed by the diffuse interface approach

Matteo Bottacchiari, Mirko Gallo, Marco Bussoletti, and Carlo Massimo Casciola

SUPPLEMENTARY MATERIAL
The lateral stress profile of fluid lipid membranes as revealed by the diffuse interface approach

Matteo Bottacchiari,[1,2] Mirko Gallo,[2] Marco Bussoletti,[2] and Carlo Massimo Casciola[2,*]

[1]*Department of Basic and Applied Sciences for Engineering, Sapienza University of Rome*
[2]*Department of Mechanical and Aerospace Engineering, Sapienza University of Rome*

(Dated: December 4, 2024)

INTERMEDIATE STEPS FOR EQ. (21)

$$
\begin{aligned}
E_{\mathrm{B}}[\phi] = {} & 4\pi k \frac{3}{\sqrt{2}} \int_0^{+\infty} \frac{f_0'^2\left(\frac{1/2-\bar{r}}{\lambda}\right)}{\lambda} \left(\frac{1}{\bar{r}} - \bar{m}\right)^2 \bar{r}^2 d\bar{r} + O(\lambda) \approx \\
& 4\pi k \frac{3}{\sqrt{2}} \int_0^{+\infty} \frac{f_0'^2\left(\frac{1/2-\bar{r}}{\lambda}\right)}{\lambda} \left(1 - 2\bar{m}\bar{r} + \bar{m}^2 \bar{r}^2\right) d\bar{r} + O(\lambda) = \\
& 4\pi k \frac{3}{\sqrt{2}} \int_{-\infty}^{1/(2\lambda)} f_0'^2\left(z^*\right) dz^* \\
& - 8\pi k \bar{m} \frac{3}{\sqrt{2}} \int_{-\infty}^{1/(2\lambda)} f_0'^2\left(z^*\right) \left(\frac{1}{2} - \lambda z^*\right) dz^* \\
& + 4\pi k \bar{m}^2 \frac{3}{\sqrt{2}} \int_{-\infty}^{1/(2\lambda)} f_0'^2\left(z^*\right) \left(\frac{1}{2} - \lambda z^*\right)^2 dz^* + O(\lambda) \approx \\
& 4\pi k \frac{3}{\sqrt{2}} \int_{-\infty}^{+\infty} f_0'^2\left(z^*\right) dz^* - 4\pi k \bar{m} \frac{3}{\sqrt{2}} \int_{-\infty}^{+\infty} f_0'^2\left(z^*\right) dz^* \\
& + 4\pi k \bar{m}^2 \frac{3}{4\sqrt{2}} \int_{-\infty}^{+\infty} f_0'^2\left(z^*\right) dz^* = \\
& 4\pi \int_{-\infty}^{+\infty} z^2 \frac{3k}{\sqrt{2}\epsilon^3} \left[f_0''^2\left(-\frac{z}{\epsilon}\right) + f_0'\left(-\frac{z}{\epsilon}\right) f_0'''\left(-\frac{z}{\epsilon}\right)\right] dz \\
& + 4\pi D_{ve} \int_{-\infty}^{+\infty} -z \frac{3}{\sqrt{2}} \frac{2mk}{\epsilon^2} f_0'\left(-\frac{z}{\epsilon}\right) f_0''\left(-\frac{z}{\epsilon}\right) dz \\
& + \pi D_{ve}^2 \int_{-\infty}^{+\infty} -m^2 \frac{3}{\sqrt{2}} \frac{k}{\epsilon} f_0\left(-\frac{z}{\epsilon}\right) f_0''\left(-\frac{z}{\epsilon}\right) dz = \\
& 4\pi \int_{-\infty}^{+\infty} z^2 \, s_{\mathrm{B2}}(z) \, dz + 4\pi D_{ve} \int_{-\infty}^{+\infty} z \, s_{\mathrm{B1}}(z) \, dz \\
& + \pi D_{ve}^2 \int_{-\infty}^{+\infty} s_{\mathrm{B0}}(z) \, dz \, ,
\end{aligned}
$$

* carlomassimo.casciola@uniroma1.it

INTERMEDIATE STEPS FOR EQ. (23)

$$
\begin{aligned}
E_{\mathrm{G}}[\phi] =& k_{\mathrm{G}} \frac{35}{8\sqrt{2}} 4\pi \int_0^{+\infty} \frac{f_0'^4\left(\frac{1/2-\bar{r}}{\lambda}\right)}{\lambda}\, d\bar{r} + O(\lambda^2) = \\
& k_{\mathrm{G}} \frac{35}{8\sqrt{2}} 4\pi \int_{-\infty}^{1/(2\lambda)} f_0'^4(z^*)\, dz^* \;+\; O(\lambda^2) \;\approx \\
& k_{\mathrm{G}} \frac{35}{8\sqrt{2}} 4\pi \int_{-\infty}^{+\infty} f_0'^4(z^*)\, dz^* \;= \\
& k_{\mathrm{G}} \frac{35}{8\sqrt{2}} 4\pi \int_{-\infty}^{+\infty} \frac{z^{*2}}{2} \left[12 f_0'^2(z) f_0''^2(z) + 4 f_0'^3(z) f_0'''(z)\right] dz^* = \\
& 4\pi \int_{-\infty}^{+\infty} z^2\, s_{\mathrm{G}}(z)\, dz\,,
\end{aligned}
$$

INTERMEDIATE STEPS FOR EQ. (26)

$$
\begin{aligned}
E_{\mathrm{T}}[\phi] =& \gamma\, D_{ve}^2 \frac{3}{2\sqrt{2}}\, 4\pi \int_0^{+\infty} \frac{f_0'^2\left(\frac{1/2-\bar{r}}{\lambda}\right)}{\lambda} \bar{r}^2\, d\bar{r} + O(\lambda^2) = \\
& \gamma D_{ve}^2 \frac{3}{2\sqrt{2}} 4\pi \int_{-\infty}^{1/(2\lambda)} f_0'^2\,(z^*) \left(\frac{1}{2} - \lambda z^*\right)^2 dz^* + O(\lambda^2) \approx \\
& \gamma\, \pi D_{ve}^2\, \frac{3}{2\sqrt{2}} \int_{-\infty}^{+\infty} f_0'^2\,(z^*) dz^* = \\
& \pi D_{ve}^2 \int_{-\infty}^{+\infty} -\gamma \frac{3}{2\sqrt{2}} \frac{1}{\epsilon} f_0\left(-\frac{z}{\epsilon}\right) f_0''\left(-\frac{z}{\epsilon}\right) dz = \\
& \pi D_{ve}^2 \int_{-\infty}^{+\infty} s_{\mathrm{T}}(z)\, dz\,.
\end{aligned}
$$
